# The Role of Metformin in Metabolic Disturbances during Pregnancy: Polycystic Ovary Syndrome and Gestational Diabetes Mellitus

**DOI:** 10.1155/2014/797681

**Published:** 2014-12-08

**Authors:** Joselyn Rojas, Mervin Chávez-Castillo, Valmore Bermúdez

**Affiliations:** Endocrine and Metabolic Diseases Research Center, School of Medicine, University of Zulia, 20th Avenue, Maracaibo 4004, Venezuela

## Abstract

Maintenance of gestation implicates complex function of multiple endocrine mechanisms, and disruptions of the global metabolic environment prompt profound consequences on fetomaternal well-being during pregnancy and postpartum. Polycystic Ovary Syndrome (PCOS) and gestational diabetes mellitus (GDM) are very frequent conditions which increase risk for pregnancy complications, including early pregnancy loss, pregnancy-induced hypertensive disorders, and preterm labor, among many others. Insulin resistance (IR) plays a pivotal role in the pathogenesis of both PCOS and GDM, representing an important therapeutic target, with metformin being the most widely prescribed insulin-sensitizing antidiabetic drug. Although traditional views neglect use of oral antidiabetic agents during pregnancy, increasing evidence of safety during gestation has led to metformin now being recognized as a valuable tool in prevention of IR-related pregnancy complications and management of GDM. Metformin has been demonstrated to reduce rates of early pregnancy loss and onset of GDM in women with PCOS, and it appears to offer better metabolic control than insulin and other oral antidiabetic drugs during pregnancy. This review aims to summarize key aspects of current evidence concerning molecular and epidemiological knowledge on metformin use during pregnancy in the setting of PCOS and GDM.

## 1. Introduction

Infertility currently affects approximately 48.5 million of women aged 20–44 years around the world [1], with severe implications in their physical and mental well-being [2]. Female fertility entails a complex array of endocrine mechanisms surrounding the integrity of the hypothalamus-pituitary-ovary (HPO) axis, which are especially important in maintenance of a healthy pregnancy, particularly due to the demands of the growing fetus [3]. Many conditions may disrupt this environment, and Polycystic Ovary Syndrome (PCOS)—an endocrine-metabolic disease that encompasses multiple hormonal alterations related to female infertility—stands out mainly due to its high prevalence, affecting 6-7% of women aged 12–45 years [4], with a worrisome 70% of women estimated to remain undiagnosed [5].

The hallmarks of this gynecoendocrine disease are disruption of ovarian steroidogenesis, giving rise to hyperandrogenemia and insulin resistance (IR) [6]. A complex IR-hyperinsulinemia-hyperandrogenemia cycle involved in the endocrine disruptions in PCOS [7] leads not only to the typical clinical picture of PCOS—featuring oligoanovulation and hyperandrogenic manifestations—but also to diverse cardiometabolic comorbidities, such as impaired glucose tolerance [8], dyslipidemia [9], hypertension [10], central obesity [11, 12], accelerated atherosclerosis [13], and metabolic syndrome [14], which can appear as a myriad of distinct metabolic phenotypes [15] including mild, moderate, and severe forms of PCOS.

Insulin resistance is an important component in the etiopathogenesis of PCOS, being associated with obesity,* acanthosis nigricans*, hirsutism [16], and early pregnancy loss [17] in these women. In addition, utilizing the HOMA-IR index as a surrogate for IR quantification, Huidobro et al. [18] reported this condition to be associated with gestational diabetes mellitus (GMD), which supports the notion that this pregnancy-related metabolic disorder may be part of the insulin resistance syndrome [14, 19]. Moreover, GDM is observed in almost 50% of pregnancies in women with PCOS [10], which has been described as an independent predictor of the former [20]. Although the consequences of PCOS are not limited to reproductive dysfunction, these implications often represent the most critical aspect for both patients and clinicians, as it conveys an increase in the risk for pregnancy-induced hypertension, preeclampsia, and preterm birth [20].

Amidst the metabolic milieu generated by PCOS, GDM appears when pancreatic *β*-cell function is unable to compensate the converging increase of both PCOS-related IR and normal gravidic IR [21]. Given the profound influence IR exerts on reproduction, it has become an important pharmacological target, associated with improvement of ovulation induction [22], prevention of endocrine-metabolic gestational complications [23], and management of GDM [24]. To this end, oral antidiabetic agents such as metformin have been proposed as a valuable tool during pregnancy [25], albeit remaining an FDA Pregnancy Category B drug [26].

The purpose of this review is to describe the pharmacology of metformin during gestation and analyze its benefits in metabolically challenged pregnancies, such as in women with PCOS and/or GDM. We have compiled several peer-evaluated studies, both prospective and cross-sectional, which aid in the description and analysis of the role of metformin during pregnancy, including animal models,* in vitro* analyses, and clinical studies. These data were organized per the following reasoning: (a) the role of IR in the development of PCOS and GDM; (b) the impact of their endocrine-metabolic derangements in pregnancy; and (c) the use of metformin in regards to PCOS and pregnancy and in GDM.

## 2. Insulin Resistance as the Key Endocrine Disruption in Polycystic Ovary Syndrome

**Figure 1:**
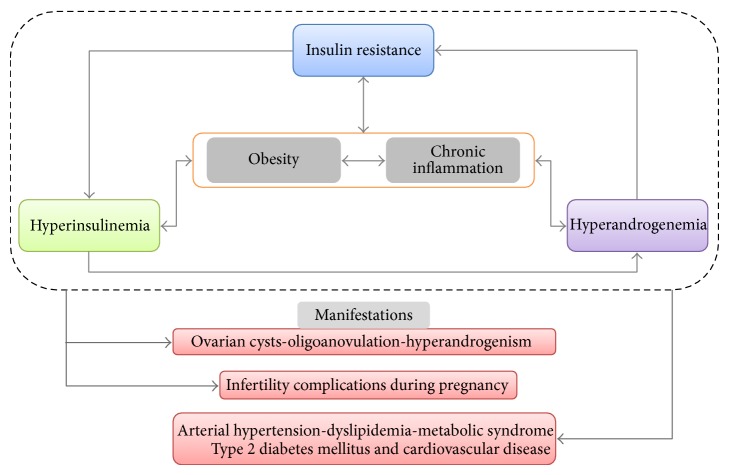
The insulin resistance-hyperinsulinemia-hyperandrogenemia cycle in Polycystic Ovary Syndrome. PCOS is dominated by three major endocrine disruptions: insulin resistance, hyperinsulinemia, and hyperandrogenemia. Although it is difficult to establish which disturbance develops first in any given case, these components are interconnected by many reinforcing mechanisms, constituting a positive feedback cycle. Furthermore, obesity and chronic inflammatory states—present in both obese and lean women with PCOS—amplify pathophysiologic pathways linked to all elements in this triad. The cycle leads to the manifestations of PCOS and infertility, complications during pregnancy, and chronic cardiometabolic comorbidities.

The etiology of PCOS is complex and multifactorial, including several endocrine disturbances, such as (a) increased pulsatile secretion of gonadotropin-releasing hormone (GnRH) and luteinizing hormone (LH), prompting theca cell hyperstimulation and androgen hypersecretion [27]; (b) nonselection of a dominant ovarian follicle, mediated by intrinsic and extrinsic ovary factors, with follicular cells hyperplasia [28]; (c) genetic predisposition to hyperandrogenemia, linked to abnormal* in utero* androgenic exposure [29]; and (d) genetic predisposition to hyperinsulinemia, also linked to prenatal androgen exposure and pancreatic *β*-cell dysfunction [30]. Although it is difficult to establish the relative importance or chronology of these and subsequent alterations, PCOS is characterized by an IR-hyperinsulinemia-hyperandrogenemia positive feedback circuit (Figure 1), where the latter component determines the majority of clinical manifestations and the diagnostic criteria for this condition (Table 1). Moreover, obesity is a very common feature in females with PCOS, which appears to magnify all previous pathophysiologic mechanisms [7].

Insulin resistance, defined as a decrease in cellular responsiveness to insulin signaling [31], triggers increased insulin secretion, a phenomenon termed “compensatory hyperinsulinemia” [32]. Although this mechanism attempts to maintain lipid, carbohydrate, and protein metabolism homeostasis, it contributes to multiple aggregate consequences, such as the cardiovascular PCOS comorbidities [33], and favors hyperandrogenemia through various pathways. In this respect, disruption of the HPO is particularly relevant: insulin has been shown to elevate GnRH and LH secretion both dose- and time-dependently [34, 35], potentially mediated through the MAPK pathway [36]. This results in increased frequency and amplitude of GnRH and LH pulse secretion, with increased LH/FSH ratio, potentiating ovarian steroidogenic alterations [6]. Other features frequently found in women with PCOS act in synergy with insulin towards enhancing LH release, including hyperleptinemia via AgRP/NPY neural pathways and kiss peptidergic signaling [37], and decreased opioidergic tone, which appears to sensitize pituitary LH-secreting cells to GnRH signaling [38]. Hyperinsulinemia has also been associated with diminished Sex Hormone-Binding Globulin (SHBG) levels, although insulin appears to be unable to directly inhibit* shbg* expression; instead, this effect depends on hyperglycemia-mediated Hepatocyte Nuclear Factor 4-*α* downregulation [39]. Lower SHBG synthesis results in increased sex hormone availability, exacerbating androgenic signaling [40].

Lastly, PCOS is also characterized by selective IR in ovarian tissue, wherein mitogenic pathways are favored while metabolic signaling is absent, yielding follicular cell hyperplasia and potentiation of steroidogenesis [41]. Several theories surround this concept, including cAMP-dependent activation of PKA with subsequent activation of Steroidogenic Acute Regulatory (StAR) protein [42], increased PI3K/Akt activity via serine phosphorylation by a hypothetical kinase in theca cells [43], and inositolphosphoglycan signaling, which appears to deviate from insulin-dependent pathways aside from being activated by the insulin receptor itself [44]. At any rate, IR-hyperinsulinemia activity leads to hyperandrogenemia, which in turn induces pro-IR structural and functional modifications in key insulin target tissues, including decreased amount of more oxidative, insulin-sensitive type I muscle fibers, and increased amount of more glycolytic, less sensitive type II fibers [40], as well as elevated lipolysis in adipocytes, favoring free fatty acid- (FFA-) mediated IR [45], perpetuating the IR-hyperinsulinemia-hyperandrogenemia feedback [7].

Although physical activity and lower caloric intake are considered fundamental lifestyle interventions [46], insulin-sensitizing agents are also a hallmark of PCOS management, with metformin being the most frequently used molecule [47]. Metformin has been described to offer significant improvement of several parameters, including Body Mass Index (BMI), LH, androstenedione, testosterone [48], DHEAS, blood pressure [49], menstrual cyclicity, fasting insulin [50], IR, dyslipidemia, oxidative stress, endothelial dysfunction [51], and several inflammatory markers [52]. This biguanide has also been reported to improve other features such as anovulation rate and acne [53] as well as BMI and LH [54] in non-IR women with PCOS. Moreover, it appears to be beneficial in both obese and lean women with PCOS [53], which may explain the persistent benefits of metformin even with several different metabotypes.

The subset of lean women with PCOS is particularly interesting. Although all PCOS phenotypes tend towards a more “apple-like” adipose distribution [55], lean subjects usually have less visceral fat [56]. Likewise, in these individuals, IR and hyperandrogenemia are predominantly related to low SHBG levels [57], with increased risk for elevated inflammation markers [58] and early vascular disease [59]. Although both lean and obese PCOS women tend to exhibit higher oxidative stress [60], they appear to behave differently regarding aging and risk of developing type 2 diabetes mellitus (DM2), which seems to be less frequent in lean women with PCOS [61]. Indeed, women who are able to maintain normal weight with aging appear to boast a healthier metabolic profile than those who do not [62]. These differences may influence the impact of metformin in each group [63]: whereas reproductive benefits are observed in both obese and lean PCOS women [64], metabolic advantages, such as lowering of proinsulin and insulin levels, are seen predominantly in the obese and overweight subset [65].

Other antidiabetic drugs have been evaluated to be applied in PCOS, particularly thiazolidinediones (TZD). Despite reports indicating these agents to be more effective than metformin at reducing IR in subjects with PCOS [66], their use remains less widespread, due to concerns of increased cardiovascular risk [67]. Indeed, despite significantly ameliorating IR, glucose homeostasis, hyperandrogenic ovarian response, and systemic inflammation [68, 69], TZD appear to induce several deleterious modifications in cardiac tissue transcriptomes, including upregulation of metalloproteinases implicated in atheromatous plaque rupture, potassium channels required for action potential generation, and genes involved in sphingolipid and ceramide metabolism [70]. Beyond these molecular findings, the impact of TZD on cardiovascular risk is also reflected in epidemiologic findings, with a higher risk of congestive heart failure in prediabetic and diabetic subjects (RR = 1.72, 95% CI: 1.21–2.42, *P* = 0.002) [71].

## 3. Exacerbation of Physiologic Insulin Resistance as the Fundament of Gestational Diabetes Mellitus

**Figure 2:**
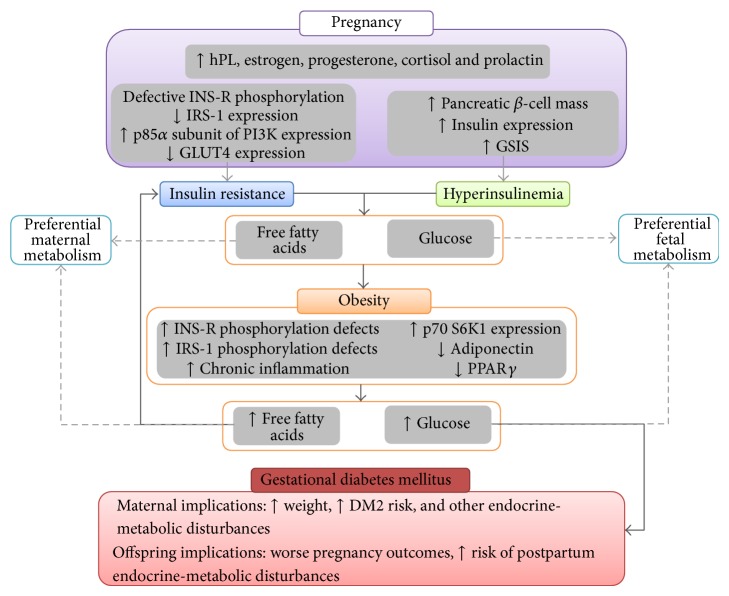
Mechanisms underlying insulin resistance in normal pregnancy physiology and gestational diabetes mellitus. Insulin resistance is a physiologic state which develops parallel to increased secretion of hPL, estrogen, progesterone, cortisol, and prolactin, principally. Although they favor IR by altering components of peripheral insulin signaling cascades, they also activate various mechanisms enhancing *β*-cell function. The result is an increased release of free fatty acids, which are predominantly metabolized by mothers, allowing for shunting of glucose towards fetal metabolism. In obesity several pathophysiologic mechanisms worsen IR in target tissues, leading to greater free fatty acid levels and dysregulation of glucose homeostasis. DM2: type 2 diabetes mellitus; GSIS: glucose-stimulated insulin secretion; hPL: human placental lactogen; INS-R: insulin receptor; IRS-1: insulin receptor substrate-1; PPAR*γ*: peroxisome proliferator-activated receptor *γ*.

Insulin resistance is a physiologic state during gestation, driven by several maternal hormones such as estrogen, progesterone, cortisol, and particularly human placental lactogen (hPL) [72]. Target cell modifications include defective tyrosine phosphorylation of the *β* subunit of the insulin receptor [73] and decreased expression of IRS-1 [74], whereas expression of the p85*α* subunit of phosphoinositol 3-kinase is increased, which interferes with heterodimeric conformation of this enzyme and thus prevents further insulin signaling [72]. Similarly, GLUT4 expression has been noted to be decreased in adipose tissue of pregnant females, significantly hindering insulin responsiveness [75]. Although the elevated serum levels of free fatty acids triggered by IR represent an important adaptive mechanism in order to increase the glucose offer for fetal metabolism, they also serve as a self-reinforcing pathway for IR (Figure 2) [76].

These pro-IR phenomena are counterbalanced by several pancreatic function-enhancing signals, which allow for the typical over twofold increase in insulin secretion during the second and third trimesters of gestation [77]. These signals include hPL, prolactin, and estrogens, all of which rise progressively and prominently throughout pregnancy [78], associated with increases in pancreatic *β*-cell mass and insulin transcription, and improve glucose-stimulated insulin secretion by promoting glucokinase and GLUT-2 expression, as well as raising glucose utilization and oxidation in pancreatic *β* cells [78]. These compensatory pathways are valuable, as they aim to maintain adequate glucose metabolism whilst allowing for increased FFA production [77]. Nonetheless, these mechanisms may be intrinsically defective or insufficient in some women, leading to the development of GDM, defined as glucose intolerance of onset or first recognition during pregnancy [79].

To this end, obesity is an important risk factor for GDM, with an OR = 2.6; 95% CI: 2.1–3.4; *P* < 0.05 [80]. Aside from enhancing all previously described pro-IR mechanisms [72], obesity favors the development of a systemic inflammatory state, with elevated levels of mediators such as TNF [81]. This cytokine is implicated in IR by allowing IRS-1 serine phosphorylation via activation of JNK and NF-*κ*B pathways [82]. Likewise, states of nutrient excess have been linked to upregulation of p70 S6K1, an IRS-1 serine kinase which induces degradation of this protein and may contribute to IRS-1 deficiency in GDM [72]. Similarly, both obesity and PCOS are associated with decreased expression of GLUT4 [83].

Another important factor is adiponectin, a proteic hormone with insulin-sensitizing activity, whose levels are decreased in obesity [84]. Although adipocytes are the primary site for adiponectin synthesis, placental production of adiponectin appears to be a paramount regulator of metabolism homeostasis during gestation [85]. Moreover, cytokines such as TNF, IFN*γ*, IL-6, and leptin have been found to modulate adiponectin and adiponectin receptor expression in women with GDM [86], harmonizing with reports associating hypoadiponectinemia with postpartum IR, *β*-cell dysfunction, and dysglycemia [87]. Expression of PPAR*γ* is also diminished, leading to subdued lipogenic pathways, favoring greater FFA release [88] and disturbance of proper lipid partition, which would enhance lipid deposition in nonprofessional tissues such as skeletal muscle, enhancing the IR cycle [7]. Other related metabolic markers have been independently associated with higher risk for GDM: the Coronary Artery Risk Development in Young Adults (CARDIA) Study [89] reported that impaired fasting glucose (OR = 4.74; 95% CI: 2.14–10.51; *P* < 0.01), hyperinsulinemia (OR = 2.36; 95% CI: 1.20–4.63; *P* < 0.01), and low levels of HDL-C (OR = 3.07; 95% CI: 1.62–5.84; *P* < 0.01) are associated with GDM risk after adjusting for race, age, parity, and birth order.

## 4. Implications of Gestational Diabetes Mellitus on Fetomaternal Health

Gestational diabetes mellitus has been noted to prevail in females with predisposition to metabolic disturbances, with pregnancy acting as stress test on endocrine physiology [90], reflected on both obesity and PCOS representing independent risk factors for GDM, as previously discussed [20, 80]. This condition entails several consequences on both mother and offspring well-being. Maternal implications consist principally of higher risk for development of DM2 after pregnancy, with approximately 10% of women diagnosed with DM2 shortly after delivery and up to 40% after 10-year follow-up [91]. Indeed, gestation may reveal or worsen preexisting defects in *β*-cell function, accelerating onset of DM2 and other related conditions [90]. This influence is present even in nonobese women with GDM, with findings of endothelial dysfunction and chronic inflammation markers—both associated with the pathogenesis of DM2, cardiovascular disease, and metabolic syndrome—in this population [92]. HOMA-IR assessment boasts promising results as predictor of postpartum *β*-cell dysfunction [93].

On the other hand, the Hyperglycemia and Adverse Pregnancy Outcome (HAPO) study [94] has demonstrated that hyperglycemia during pregnancy—even in nondiabetic ranges—is associated with increased birth weight and elevated cord blood C-peptide serum levels. GDM is related to greater risk of macrosomia, shoulder dystocia, birth injuries, neonatal hypoglycemia, hypocalcemia, hyperbilirrubinemia, respiratory distress syndrome, and polycythemia [95], as well as teratogenesis, particularly in obese subjects [96]. Furthermore, elevated cord-blood insulin concentrations are linked to glucose intolerance in offspring, and children exposed to GDM appear to display various metabolic disturbances well into childhood, including higher blood pressure and lower HDL-C [97].

These epidemiological data obey profound disruptions in embryonic and fetal metabolism, and numerous hypotheses attempt to explain this panorama. The theory of fuel-induced teratogenesis was first outlined by Freinkel [98], who proposed fuel excess and overgrowth to be the pathogenic basis of maternal hyperglycemia. This notion is founded on findings of maternal hyperglycemia-induced enhancing fetal insulin secretion, potentiating tissue growth—macrosomia—via fetal IGF-1 [99]. Alternatively, Hales and Barker [100] have propelled the thrifty phenotype theory, suggesting* in utero* malnutrition to bear a strong influence on postnatal risk of obesity, cardiovascular disease, and DM2, and even risk of PCOS and future pregnancy complications [101]. These premises are complemented by the concept of metabolic memory, related to endocrine-metabolic reprogramming of offspring amidst the diabetic environment during pregnancy [102]. This notion encompasses fetal inflammation, blunted myogenesis, oxidative stress, and disruption of immune system tolerance, among various other alterations [103]. Likewise, fetal exposure to diabetes appears to modify hypothalamic functionality in animal models, associated with hyperphagic behavior and obesity-proneness after birth [104].

AMP-dependent kinase (AMPK), a classic target of metformin action, may be an important mediator in this context [105], as it intervenes in processes such as lipogenesis via inhibition of acetyl-CoA carboxylase [106], myogenesis through the modulation of myocyte enhancer factor 2 [107], cell cycle [108], and appetite pathways [109]. Animal models have shown that metformin-induced AMPK activation yields beneficial effects over embryonic implantation [110], fetal inflammation [111], maternal liver function [112], and pregnancy outcomes [113]. Notwithstanding that these and other molecular pathways remain under research and certain aspects require further characterization, metformin has proven to beat the test of time, standing as a promising recourse in many circumstances, including GDM.

## 5. Metformin Pharmacokinetics during Pregnancy

**Figure 3:**
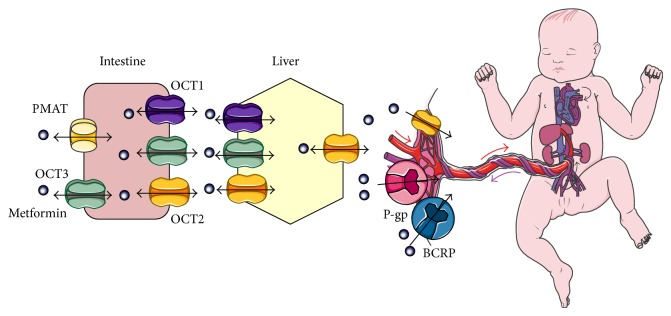
Absorption and distribution of metformin during pregnancy.

Uptake and distribution of metformin towards the circulatory system requires the participation of bidirectional transporters located in the intestine and liver [114, 115]; see Figure 3. In the apical membrane of enterocytes, PMAT (Plasma Membrane Monoamine Transporter) and OCT3 (Organic Cation Transporters) mediate absorption. Mobilization of the drug towards the liver requires OCT1, OCT2, and OCT3, while OCT2 is needed in order to reach the bloodstream, kidneys, and excretion [116]. Renal clearance of metformin increases during mid (723 ± 243 mL/min, *P* < 0.01) and late pregnancy (625 ± 130 mL/min, *P* < 0.01) [116], relating to a concentration of the drug in umbilical cord blood at time of birth between undetectable levels and 1263 ng/mL. Placental tissue expresses OCT2 transporter, yet under strict epigenetic control [117, 118], underlying ample interindividual differences in this aspect. However, other transporters are also involved in drug efflux through the placenta. Reflecting the high protectiveness of the human syncytiotrophoblast regarding the fetus, this tissue has been described to express a series of transporters in the apical membrane, such as P-glycoprotein (P-gp), Multidrug Resistance-Associated Protein 1 (MRP1), and Breast Cancer Resistance Protein (BCRP) [119–122], with metformin being transported mainly via P-gp (58% ± 20%) and BCRP (25% ± 14%) [119]. Competition between this biguanide and other drugs can also limit the exposure of the fetus, further limiting the presence of toxic concentrations during pregnancy.

Animal studies using dosages up to 600 mg/kg daily have failed to report evidence of teratogenic effects [123] and extremely high dosages between 900 and 1500 mg/kg daily failed to induce carcinogenicity [124]. Furthermore, in 2003 Gutzin et al. [125] reported their results concerning first trimester exposure, ascertaining no higher rates of major malformations with an OR of 1.05 (95% CI: 0.65–1.70), while neonatal death rendered an OR of 1.16 (95% CI: 0.67–2.00). Likewise, Gilbert et al. [126] conducted a meta-analysis on 8 studies concerning fetal malformations associated with metformin use during pregnancy, indicating this drug to yield an OR of 0.50 (95% CI: 0.15–1.60)—rendering a minor protective effect. Finally, the pooling analysis showed that the control group had a malformation rate of 7.2%, compared to 1.7% in the metformin group [126], strongly supporting metformin's safety during pregnancy.

Concerning breast milk-related exposure [127], it has been confirmed that metformin can be detected at ranges between 0.13 and 0.28 mg/mL, equivalent to <0.5% of the mother's weight-adjusted dosage [106]. Other reports have quantified metformin in breast milk at 0.28–1.08% [128] and 0.18–0.21% [129] of maternal dose. Placental partition coefficient for metformin has been calculated at 36.3%, with a cord plasma concentration of 0.1–2.9 mg/L during labor [130]. Such findings confirm that neonatal exposure to metformin is actually quite insignificant, and it is not related to glucose abnormality in infants, granting safe use before, during, and after pregnancy [128–130].

## 6. Metformin Use in Pregnant Women with Polycystic Ovary Syndrome: Different Outcomes, Different Efficacy

Because infertility is one of the main consequences of female reproduction in patients with PCOS [4, 5], ovulation induction remains the most common intervention during fertility counseling. Current guidelines heavily promote lifestyle modifications and support clomiphene as the first-line agent for ovulation induction, while recognizing that complementation with metformin improves ovulation and pregnancy success [131], as reported by Lord et al. [22] in their meta-analysis concerning effectiveness of this antidiabetic drug in achievement of ovulation in 15 trials involving 543 participants. This yielded an OR of 3.88 (95% CI: 2.25–6.69) for metformin alone and 4.41 (95% CI: 2.37–8.22) for metformin combined with clomiphene. In addition, the results from Khorram et al. [132] showed that two-week treatment with insulin reduced insulin levels and IR while improving SHBG levels and clomiphene-induced ovulation. In regards to metformin and gonadotropin use, Palomba et al. [133] reported that the biguanide improved live birth rates (OR = 1.95; 95% CI: 1.10–3.44; *P* = 0.020) and pregnancy success (OR = 2.25; 95% CI: 1.50–3.38; *P* < 0.0001).

Early pregnancy loss (EPL) is defined as the interruption of pregnancy before the 20th week of gestation [134]. Although chromosomal abnormalities are the principal cause of EPL [135], they are uncommonly reported in women with PCOS [136]. It has been proposed that endocrine disruptions may play a role in EPL, with elevated androgens being associated with EPL in women with PCOS, and with recurrent EPL in women with and without PCOS [21]. Additionally, several endometrial molecular alterations have been described during implantation in PCOS: (a) androgen-dependent suppression of glycodelin [137], a cell-adhesion molecule involved in endometrial receptivity [138]; (b) IR-hyperinsulinemia can also diminish glycodelin expression, alongside IGFBP-1, key molecules for endometrial preimplantation maturation [139]; and (c) a hypofibrinolytic state due to increased synthesis of plasminogen activator inhibitor-1 (PAI-1), which has been found to be an independent risk factor for EPL in PCOS [140]. In this context, PCOS patients prescribed with metformin have lower pooled odds ratios for EPL (OR = 0.32, 95% CI: 0.19–0.55) and preterm birth (OR = 0.30, 95% CI: 0.13–0.68) [141], suggesting that this treatment can reverse the impact of PCOS on implantation success observed in this gynecoendocrine disease.

Other benefits have been attributed to metformin throughout gestation in women with PCOS, but perhaps one of the most important ones, is the 40% reduction of new-onset diabetes in high risk individuals as reported by Salpeter et al. [142]. In their meta-analysis using 31 trials and 4,570 subjects, the resulting pooled OR was 0.6 (95% CI: 0.5–0.8), with an absolute risk reduction of 6% (95% CI: 4–8) during a period of treatment of 1.8 years [142]. On the other hand, Nawaz et al. [143] have described decreased prevalence of fetal growth restriction and increased live birth rates, as well as an absence of intrauterine deaths or stillbirths, in women taking metformin during pregnancy, in line with claims of metformin being unrelated to teratogenicity [144].

Nevertheless, metformin during pregnancy appears unable to significantly reduce rates of preeclampsia and preterm birth in subjects with PCOS. A randomized, placebo-controlled, double-blind, multicenter study by Vanky et al. [145] found that preeclampsia prevalence was 7.4% in the metformin group and 3.7% in the placebo group (3.7%; 95% CI: −1.7–9.2; *P* = 0.18), whereas preterm birth prevalence was 3.7% in the metformin group and 8.2% in the placebo group (−4.4%; 95% CI: −10.1–1.2; *P* = 0.12); the inefficacy of metformin at preventing preeclampsia may be due to the complex etiopathogenesis of this disease. Data from Stridsklev et al. [146] support this phenomenon, in which reporting metformin treatment did not affect uterine artery flow during gestation, while also describing an association between uterine artery flow and androgens, highlighting the complexity of the mechanisms underlying placentation, conservation of uterine artery flow, and vessel compliance [147, 148].

Indeed, despite several mechanisms related to IR-hyperinsulinemia being involved in the etiopathogenesis of preeclampsia—chronic systemic inflammation, increased sympathetic tone, and vascular smooth muscle growth [149]—metformin may be unable to effectively modify the pathogenic root of this disease, which is faulty placentation [150]. Similarly, although metformin's effects may aid in prevention of preterm birth by ameliorating oxidative stress and chronic inflammation [151], various elements underlying preterm labor may escape the reach of metformin's activity, including the most common factors associated with this condition—defective placentation, intrauterine infection, and maternal immunologic receptivity [152].

Still, metformin seems to offer other benefits to offspring of women with PCOS even in the postnatal period. In this scenario, metformin throughout pregnancy has been associated with diminished neonatal hypoglycemia [153], as well as normal growth and motor-social development in the first 18 months of life [154]. Likewise, the growth and motor-social skills of breast-fed children of women with PCOS taking metformin have been demonstrated to be similar to those of formula-fed infants, with no abnormalities [155].

## 7. Metformin in Pregnant Women with Gestational Diabetes Mellitus: Challenging Insulin as the Go-To Therapy

Although insulin therapy has been considered the best management option for GDM, recent evidence diverges from this precept. The first major trial concerning the use of metformin and/or insulin during pregnancies complicated with GDM was the metformin in gestational diabetes (MiG) [156], whose goal was to determine the effects of either drug on prevention of fetal hyperinsulinemia and promotion of lower maternal glycemia. This research group ascertained metformin (500–2500 mg/day) with or without supplemental insulin not to be associated with higher perinatal complications, in comparison to insulin alone [157], findings later corroborated by Silva et al. [158]. Furthermore, patients tend to prefer metformin over insulin as treatment schemes and would rather be prescribed such drug if possible [156]. Likewise, metformin use during pregnancy failed to adversely affect maternal lipid parameters, C-reactive protein levels, or birth weight [159].

After this emblematic trial, several other studies have supported the effectiveness of metformin in GDM. Niromanesh et al. [160] conducted a randomized controlled trial with 160 pregnant patients with GDM, 80 of them treated with metformin (500–2500 mg) and the rest with insulin NPH (0.2 U/kg bedtime) and regular (1 U per 10 mg/dL over). Results revealed metformin to reduce rates of macrosomia and maternal weight gain. Additionally, Rowan et al. [161] also ascertained a decline in macrosomia and preeclampsia rates and suggested glycemic goals in GDM should be more rigorous. Metformin in GDM has also been described to lower incidence of surgical delivery [162]. Notably, these effects are observed even in spite of lowering of vitamin B12 [163], a recognized side effect of the drug [164].

Although various oral hypoglycemic agents—aside from metformin—are known to confer adequate metabolic control during pregnancy compared to insulin [165], metformin seems to be the superior choice, offering better control than glyburide, as reported by Silva et al. [158]. This research group has also reported newborns from mothers treated with metformin to obtain lower weight (3193 g versus 3387 g; *P* = 0.01) and ponderal index results (2.87 versus 2.96; *P* = 0.05) as well as less maternal weight gain, in women with GDM, when compared to those treated with glyburide (10.3 kg versus 7.6 kg; *P* = 0.02) [166], possibly reducing probabilities of other weight-related complications, such as preeclampsia. On the other hand, data on TZD use during GDM is relatively scarce, and trials conducted to date are considered insufficient to definitively establish these drugs as safe during pregnancy [25]. In this context, PPAR*γ* has been noted to be key in embryonic development [167], and TZD administration during pregnancy has been associated with impaired fetal development [168], with this drug class remaining within the FDA Pregnancy Category C [169]. Therefore, further research is needed to explore the role of TZD in pregnancy and GDM.

Beyond evidence supporting metformin use in GDM, a key issue regarding pharmacological management of this disease is the prediction and selection of the best suited alternative (insulin alone, metformin alone, or both combined) for each specific patient. Insulin remains the most recommended option in mild cases of GDM [170] and in women with elevated BMI [171]. Indeed, in women with GDM, HOMA-IR values 1.29–2.89—interpreted as decreased insulin secretion—have been proposed to indicate a requirement of insulin therapy, whereas values >2.89 are thought to underline insufficient compensation of IR, rendering insulin-sensitizing agents more adequate [172]. Likewise, women with GDM and a fasting glucose result from oral glucose tolerance test below 93.3 mg/dL have displayed a probability of favorable pharmacological response of 93% to metformin [173]. On the other hand, early detection of GDM is a predictor for supplemental insulin treatment in women initially treated with metformin [174], as well as older age and elevated serum fructosamine concentration [175].

## 8. Concluding Remarks

Pregnancies complicated with GDM or with history of PCOS are a challenge for both obstetricians and endocrinologists, representing a halfway point where these specialties merge and highlighting the importance of multidisciplinary prenatal management. In our experience, we have observed that patients with PCOS who continue with metformin treatment throughout pregnancy and those who receive this drug as a pharmacological intervention in GDM yield better pregnancy outcomes and a better postpartum metabolic prognosis for both mothers and their offspring.

Nevertheless, further studies are needed to uncover and elucidate the benefits and shortcomings of metformin in this context, in both molecular and epidemiological fields. Ongoing studies concerning these issues include the Metformin to Prevent Late Miscarriage and Preterm Delivery in Women With Polycystic Ovary Syndrome Trial (PregMet2) [176] and the Metformin Treatment in Gestational Diabetes and Noninsulin Dependent Diabetes in Pregnancy in a Developing Country Trial (migdm&t2dm) [177] as well as additional data from the MiG trial, among many others. Indeed, the future appears compelling and exciting in this aspect, with these sources promising valuable information which may reshape and refine views on metformin use during pregnancy.

## Figures and Tables

**Table 1:** Diagnostic criteria for Polycystic Ovary Syndrome.

	Clinical or biochemical hyperandrogenism	Oligo/anovulation	US finding of polycystic ovaries^*^
NIH, 1990 **BOTH** of the following:	+	+	
ESHARE/ASRM, 2003 **ONLY 2** of the following:	+	+	+
AES, 2006 **ALL 3** of the following:	+	+	+

NIH = National Institute of Health of the United States; ESHRE = European Society of Human Reproduction and Embryology; ASRM = American Society of Reproductive Medicine; AES = Androgen Excess and PCOS Society.

All sets of criteria require the exclusion of other etiologies such as congenital adrenal hyperplasia, androgen-secreting neoplasms, and Cushing's syndrome, among others.

^*^Ultrasound polycystic ovaries defined as the presence of ≥12 follicles of 2–9 mm width; or an increase in ovarian volume (>10 mL) in at least one ovary, in women not consuming oral contraceptives.
